# Every Story Is Different: Experiences With Body Changes Related to Cancer

**DOI:** 10.3389/fpsyg.2022.831811

**Published:** 2022-05-23

**Authors:** Linda Cole, Julie Easley, Leslie Grightmire, Ellil Mathiyan Lakshmanan, Sharon J. Matthias, Karen McBoyle, Emily Piercell, Amelia Purdy, Nancy Schneider, Richard J. Wassersug, Rosemary Martino, Margaret I. Fitch

**Affiliations:** ^1^Independent Scholar, Toronto, ON, Canada; ^2^Horizon Health Network, Fredericton, NB, Canada; ^3^Independent Scholar, Peterborough, ON, Canada; ^4^Ostomy Association of Singapore, Singapore, Singapore; ^5^Matthias Inc: Connecting for Innovation and Advancing Societies, Edmonton, AB, Canada; ^6^Independent Scholar, Kingston, ON, Canada; ^7^YouByMia Photography, Toronto, ON, Canada; ^8^Independent Scholar, St Walburg, SK, Canada; ^9^Independent Scholar, Vancouver, BC, Canada; ^10^Department of Speech Language Pathology, Rehabilitation Science Institute, University of Toronto, Toronto, ON, Canada; ^11^Bloomberg Faculty of Nursing, University of Toronto, Toronto, ON, Canada

**Keywords:** body image, cancer, patient/survivor perspectives, survivorship, quality of life

## Abstract

One of the important aspects of stakeholder engagement in cancer care and system planning is hearing from individuals who have been diagnosed with cancer about the impact of the diagnosis and treatment on their lives. Hearing stories from the perspectives of cancer survivors offers opportunity to gain new insight and understanding about experiences of being diagnosed and treated for cancer. This article presents ten short narratives about survivors' perspectives on body image and cancer. Each story is unique but, taken together, the picture they create is one of facing challenges, discovering personal resilience, and moving forward to engage in living. The stories emphasize the importance of communication and support from healthcare providers and understanding needs for a person-centered cancer care system.

## Introduction

A significant development over the past decade in the cancer care system has been the growth in engagement with stakeholders (Lowe et al., 2021) and understanding more from them about what it means to have a person-centered philosophy driving care delivery (American Geriatrics Society Expert Panel on Person-Centered Care, 2015; Sharma et al., 2016; Eklund et al., 2019). One of the important aspects of stakeholder engagement is hearing from individuals who have been diagnosed with a disease, and experienced its treatment, about their perspectives and the impact of the diagnosis and treatment on their lives (Nolte et al., 2020; Fares et al., 2021). As the cadre of survivors has grown, an increasing number of individuals are coming forward and expressing a desire to help improve care provision and support for people facing cancer (Stover et al., 2019; LeClair et al., 2020). Hearing the stories from survivors offers an avenue to continue the journey as health care providers toward truly providing person-centered care (Frank, 2018).

Stories are vital to human experience. They are vehicles for connecting, offering a means of sharing, and interpreting experiences (Calman, 2001; Woods, 2011). They help us understand the world around us and are universal bridges for building relationships and sharing cultures. Hearing stories can enhance understanding of the experiences of others and aid in linking our thoughts and feelings. Stories can capture attention, invite conversations, and inspire the imagination for change. This article was written from a stance of valuing stories, of listening to the narrative of one, and embracing the importance of hearing stories directly from the voice of a person who has lived through an experience.

The series of stories or short narrations below are from cancer survivors about living with body image changes following cancer treatment. The stories were contributed by each of the authors working collaboratively to produce this perspectives manuscript. The stories illustrate various types of bodily changes these individuals experienced from their cancer treatment and what it has been like to live with the changes. Some changes have been temporary, and some are permanent, some visible and some concealed. The short narrations do not include all cancer types, all ages, or attention to every type of change or characteristics which might influence reactions or coping. The short narrations cannot tell the whole story for each person, but they offer a vantage point for gaining a deeper understanding about living with changes in body image. Collectively, they offer an emergent picture of the complexity inherent in the wholeness of human response. Individuals have more than one way to be in the world and to cope with its realities.

Collectively the stories paint a picture which can help us appreciate how body image is not a unidimensional concept. Living with bodily changes following cancer or its treatment is challenging and not easily separated from other aspects of life. The changes have impacts beyond the physical and are intermingled with emotional, practical, and spiritual aspects of human response.

## The Stories

### Amelia

I was diagnosed with breast cancer in August 2020, 2 days after turning 36. I had a single mastectomy and, luckily, didn't undergo chemotherapy or radiation. In my situation, the negative long-term effects of them outweighed the benefits. I'm also doing hormone therapy to help prevent a recurrence and that has affected me significantly. I'm in medically induced menopause, which has been really tough physically and emotionally. I get hot flashes, migraines, insomnia, joint pain—it makes me feel older than I am. It's such a big change. My joint pain, for example, can get very painful. If I'm out in the cold for too long, my hands can feel swollen. The hot flashes are brutal—they happen all day and are uncomfortable and make me feel clammy and sticky. And having insomnia has been physically and mentally draining.

Along with my single mastectomy, I had 3 lymph nodes removed. That has left my arm and chest weak and numb. It makes it hard to exercise and I find that I'm nervous to do so. I'm definitely not exercising as vigorously or often as I should. I'm worried about hurting my arm even though I know it needs to be strengthened.

I decided to stay flat on the one side after my mastectomy. Although I'm so glad about my choice, I find having one breast can feel weird. I have to wear a bra for the other side and clothes don't fit right. I am very conscious of only having one. When I'm out, I wonder if people will notice and what they'll think. But I've learned to tune that out and do my best not to care. I give myself pep talks a lot about it—who cares what they think? My body is amazing!

My husband is totally fine with however I look; he is very supportive and has told me on numerous occasions that he doesn't love me because of my breasts. I'm so lucky to have that support. But my daughters, who are 7 and 5, have struggled, which can be hard on me. They see my body and tend to be uncomfortable because it looks so different. I tell them it's just my body; everyone has a different body and that's what makes us beautiful. I explained that I was sick and needed to have my breast removed. They understand that, but it's taking them time to adjust.

I have decided to have my ovaries and my other breast removed as a preventive step. I am going to stay flat afterwards. When I was first diagnosed, I wanted a bilateral mastectomy with reconstruction. After finding the lump, my breasts felt totally foreign to me, and I just wanted them both gone. Unfortunately, I didn't have time to have reconstruction right away. I would need to have my breast removed and reconstruction at a later date. With that in mind, I had to decide on surgery. To help with my decision, I looked at Google images to see what the scars might look like, and the photos were just too much; I was so scared of the scars and worried I wouldn't be me if I didn't look like me. The surgeon didn't think a double mastectomy was necessary; he said I was overreacting and that I just needed a lumpectomy. But it was ultimately my decision—my body!

In the end, my surgeon and I agreed that I would have a single mastectomy and have the other side removed later. I am so glad I went with that decision. Now, I am totally comfortable with the scars and the idea of being flat. I actually feel more confident now, which is a huge surprise.

What helped me, and what I would suggest to others, is to take photos. I wanted to share photos of myself and my scar for other women going through a similar diagnosis. I wanted them to have better photos than the ones on Google. So, my friend did a photo session with me 4 weeks post-operation. I truly thought I was going to hate the photos; they were simply for others, not for me. But when I saw the photos, I cried. They were so beautiful. It was such a healing experience for me.

I've taken lots of photos since that session and have found it empowering. I'm honoring my body and what it's gone through and continues to go through.

This experience has taught me the importance of advocating for yourself. Breast cancer can happen at any age. Young women need to know the signs and be taught proper breast health. So often when young women do find a lump and seek help, they're told, “You're too young; it's just a cyst or a blocked milk duct” (which is initially what I was told). It's so frustrating. These women go home thinking they're fine, and then down the road have a delayed diagnosis and precious time wasted. So you have to push: demand tests and investigation. It's always better to know than not know. And remember that whatever decision you make is the right one for you. It's your body and you have to live with it. Do what's best for you.

### Emily

I was diagnosed with stage 3, triple positive breast cancer with lymph node involvement, the summer after I graduated from law school. I was 27, and on August 26, 2015, I had to put my career on hold to endure a year of grueling treatments. I had 5 months of chemotherapy, a double mastectomy with immediate reconstruction, 25 radiation treatments, a year of Herceptin treatments, three other surgeries to fix my reconstruction, and am currently still doing hormone therapy for another 5 years.

My cancer treatment started with chemotherapy. Chemo took all of my hair (who knew nose hair had a purpose), made me physically weak, and gain weight to the point that a different person was staring back at me in the mirror. When I look back at photos during this time, I don't recognize myself because I am so swollen from the steroids. The day before my last chemo session I finally lost my eyebrows and eyelashes, which made me really look like a cancer patient. This was a hard day for me because I had beautiful, long eyelashes which became a part of my identity. They still haven't grown back in the way they used to be.

During my double mastectomy, tissue expanders were inserted to create space for my eventual implants. After two more reconstructive surgeries, my implants failed because the skin had died from the radiation. My plastic surgeon tried cutting the dead skin and stitching it back together, hoping the skin would heal, but it never did. Once the stitches came out, I had a hole in my breast and every day it would get bigger and you could see the implant. I had an open wound for months and had to clean and bandage it up daily. I had to monitor myself for signs of infection (which happened a couple times), so a thermometer became my most important accessory. This led me to have the DIEP Flap surgery.

Those 4 months, when my implants had failed and I was weighing my options on next steps, were very hard emotionally. We were no longer dealing with actual cancer in my body and my support system didn't understand why I kept having surgeries. I'm as happy as I can be with reconstructed breasts without nipples. I now have so many scars—huge round scars around my whole breasts, a scar from literally hip to hip that looks like my stomach is always smiling, a port scar just underneath my collarbone and a concave scar in my armpit from where my lymph nodes were taken out.

After my mastectomy it took me a long time to be able to even look at myself in the mirror, let alone touch my scars. A few months after my mastectomy I started radiation and from day one you are supposed to moisturize the radiated area a few times a day. I had to get my husband to do it for me because touching myself made me feel sick. Eventually, I mustered up the courage to do it myself, but it took a bit of time.

Dealing with body image is a daily process. Most of the time I'm proud of my body and everything it has been through, but I wish I didn't have a forever “muffin top” that will be there no matter how much I exercise or such misshapen breasts. Every time I look in the mirror without my clothes on, it is a reminder that I am not a “normal” 33-year-old anymore. I've lived so long without nipples that sometimes I forget breasts are supposed to have nipples. It took me a while to be intimate with my husband without a shirt on and I still haven't felt sexy in this new body. I am also unable to do things physically that I was able to do before cancer and surgeries. For instance, I was really proud of my push-up abilities and was able to do over twenty with my feet elevated. Now, I'm lucky if I'm able to do three from my feet instead of my knees.

I'm now 6 years from my cancer diagnosis and cancer still has a huge impact on the decisions I make in my life, like fertility. I still have five more years of hormone therapy. Do I pause it to have a baby? What about my fear of recurrence and leaving my future child without a mother? Or what about passing it on to my child?

Sometimes I still have trouble trusting my body and see myself as the weak cancer patient. I'm still working through my fears and anxieties with a psychologist which really helps. And I know that most of the time I am a strong and resilient person, but I miss the days when things weren't as complicated. But, through my cancer diagnosis I have found what I'm good at—using my experience as an AYA with breast cancer to help others going through it. One thing I would say to others diagnosed with cancer is, “With time, cancer won't be the first thing you think about when you wake up.”

### Ellil

I was diagnosed 10 years ago when I was 52. I had two primary cancers—rectal and testicular. I had surgery (laparoscopic abdominoperineal resection), radiotherapy (28 cycles), and chemotherapy (8 cycles). In the beginning I was shocked and did not believe this was happening to me. I felt despair, anger, and anxiety—all of these reactions. I was worried about how I was going to live day-to-day and go to work and socialize or play any sports. I could not imagine how I was going to be able to live a normal life. I was active in sports and used to box, fence, and play squash. I could not imagine how I would be able to keep doing these.

After my surgery I had an ostomy—a permanent colostomy—and that changed the way I passed stool. So, I had to learn how to manage that—change the bag, care for the stoma itself. Because it was an end colostomy, I do not have any dietary restrictions, so that made it a lot easier. It was explained to me before the surgery that I would have an ostomy, so I expected it.

I did feel differently about my body afterwards, and I did not want to show the ostomy to anyone else, perhaps for about the first 6 months. I was actually ashamed, having to collect my stool in a bag, and didn't want anyone else to know about it. After that, I accepted that the stoma was necessary because of the surgery needed to treat my cancer, and acceptance came with it. Also, I checked all the boxes when it came to risk factors for colorectal cancer, so I couldn't really ask why me. It was more a case of why not. That made me realize it was no use dwelling on the past and decided to move forward with my life with my new friend, my stoma. And so, I adjusted quickly, and got on with it. Subsequently, I had no qualms about showing my body with my stoma bag. I guess you could say I adjusted quickly and felt acceptance about my situation. I do not feel it was a major impact on me. Now I am able to do a brisk walk, do Zumba, and dragon boating. For other people who know about the situation, having the ostomy, they have been very accepting. I think because I manage it quite well in most situations and surroundings, they have no problems with it.

For the entire first year, I only had support online, and from Colostomy UK. They sent me a postage-paid package all the way from the UK. And I joined their online support group, which I found very useful. I especially liked the positive patient stories, which lifted the doom and gloom which I was under when recovering at home. Also, the sheer number of products and accessories available for living with a stoma reassured me that I could manage my condition. And after that, I was introduced to a support group in one of the major hospitals here. Since then, I have been very involved in setting up support groups in other hospitals and co-founded the Ostomy Association of Singapore to provide psychosocial and other kinds of support to help improve the quality of life for ostomates in Singapore.

The advice I would share with others who are facing this situation would be to seek out a support group. It is possible to live a near normal life with a stoma and the conversations you have at the support group and the advice the others have to share is very helpful.

People are wired differently. Not everyone will be quick to accept having an ostomy and get on with their lives. We need to let them know it is OK and allow them to take as long as they need to come to terms with it. But in the meantime, we need to help them with the areas that are important to them—directly or indirectly through other agencies. There is always hope and there is a lot of life after a cancer diagnosis.

### Julie

I was diagnosed just over 20 years ago, when I was 23, with Hodgkin's lymphoma. I had chemo, radiation and surgery to remove lymph nodes. I feel so fortunate to be here as a 20-year survivor, but I would say my feelings and my relationship with my body have changed over time.

The moment I was told my diagnosis, my very first thought was, “I am going to lose my hair!” It seems so silly to me now. It was not about potentially dying, it was about my hair. I'm not a vain person, but at that time my hair was my identity. I had a head full of ringlets and was always known as “the girl with the curly hair.” Losing hair was a visible sign of cancer and for me a loss of who I was.

The hair came out after my second chemo and it was so messy. I decided to have a head shaving party and invited everyone who I thought would be shocked seeing me without hair. I did it in an almost ceremonial way. My biggest fear was how others would feel and react. Later, I always kept my hat on and would ask them, “Are you ready?,” before I took it off. Not that I was ashamed, but I did not want to see the look of shock and sadness in their eyes. I felt so lost at first. I was no longer “the girl with the curly hair,” I became “the young girl with cancer.”

I also had scars, but I could hide them under my clothes. I was self-conscious about them and thought they were ugly, a constant reminder of cancer. I thought if people saw them, they'd comment, and I did not want to talk about them. I did not want to stand out any more than I already did.

But now, my feelings have changed about those scars. As much as the scars have healed and softened over time, so have my feelings toward them. I embrace them as a sign of empowerment, a sign of strength. I am proud to be here, for my resilience through all of this. I would say my view of my body is more positive now than it was at the time of the diagnosis. I am proud of my body for persevering. I am proud of my scars. But that attitude took time and effort to work through over the years.

At the time, there was no one else my age with cancer in my area and very few supports. I went to see about wigs, and it was “just a wall of gray wigs.” I went to a support group and all the women were older, they had already gone through so much of their lives and the things I had yet to experience. I couldn't relate to anyone. Not the people with cancer, and not the friends my age without cancer.

When I was first diagnosed, I was so naïve and did not know a thing about cancer. I had never even set foot in a hospital before. I had no idea what to expect. I discovered there were many other issues I had to face—like loss of fertility, menopause, finances, and social isolation. Losing my hair became the least of my worries. There were even times when I didn't mind being bald, particularly in the heat of summer. I'll never forget the amazing feeling of the cool rush of water on my scalp when I would swim in the river. For the first time in my life people noticed other things about me, not just my hair.

In the end, I had braced myself for the physical changes, but not really for the emotional changes and the ever-changing relationship with my body. I had to learn to trust my body again. My diagnosis was delayed, so I feared every new lump or cough. I had to learn to be well again. Long term, I am now going through a new phase. Some new side effects from treatment seem to be emerging. It's eye opening. I feel so far removed from the original experience and have not thought about recurrence for a while. I don't know what's normal any more for someone my age, after years of being monitored and on such high alert about my health. I feel I am walking a fine line between trusting my body and not wanting to miss something again.

It's big hill to climb, dealing with the changes to your body, but my feelings about them, the scars, the different hair that grew back, have shifted. I am still known to some as “the girl who had cancer”—but now I am proud of it and the way I coped. I am not saying it was not a struggle, but what I have learned, the power I feel now, I feel I can use for good.

### Karen

I was diagnosed 5 years ago with cancer at the base of my tongue on the right side, with spread to the lymph nodes on both sides. It really was devastating when I heard, and I was only 63. I had treatment with chemotherapy and radiation for 6 weeks, which really was the worst thing ever. I was really sick from the side effects of radiation and eventually could not eat.

I do not have any permanent physical changes. But during the treatment I had a PEG tube for nutrition, and redness and peeling of my skin on my lower face and neck because of the radiation. I had hair loss on the lower part of my head and noticeable weight loss, though not to the point of me being fragile.

Because I could not eat, I ended up with a PEG tube, and the nausea was so bad I could hardly stand the smell of food. I really had decreased energy and my endurance was way down. It all gradually came back after treatment finished, but at the time it meant I was not able to do what I enjoyed in my life. I would say, though, there was one permanent change, and that was my mental state. My priorities changed in what as important or not, the values in life, and a more “self” centered attitude.

During the treatment, with the fatigue and little endurance, I really could not do anything I enjoyed. I was not able to travel from the city to our permanent home (which is a 4-h drive away) and enjoy it. I had little energy to be with my young grandchildren. So, I was not able to be where *I Wanted* to be. With the low energy I could not go golfing or hiking. And of course, I could not eat—and I love eating and trying different foods.

I was upset about all the changes, but I knew I had to have them. I expected them because the team had told me about them. I knew there was a good chance of recovery, as I was healthy to begin with, but I knew that changes could happen with the treatment I had to have. I suppose if I had to have extensive surgery and had a lot of changes to my face, it would be different. I am a physiotherapist by profession and remember treating a lot of head and neck cancer patients with extensive facial changes. That was the way it was then with the available treatments. And I even remember saying, “I do not know what I would do if I got head and neck cancer.” And here we are!

I really did not think too much about how I looked, I am not that type of person. But I did wonder if I would ever get back to doing what I loved after my body failed me. I knew what I needed to do, what was likely to happen, and I was doing all I needed to do to get better. I really did not dwell on thoughts about the physical changes. I guess, though, it did cross my mind to wonder how I would deal with things if speech and swallowing issues turned out to be permanent. But I also thought if that turned out to be the case, I would deal with it because of the type of person I am. I mean, after all, the alternative is not great.

I was really sick at one point and needed the PEG tube, but it was not obvious to others. I suppose others might have reacted to the redness and peeling skin. But I really was not out socially then as I was guarding my immunity. I guess I would have just explained it to them, if they reacted, so they would be comfortable.

My body is different now. I am back to golf, but I have to watch too much arm movement because it hurts my neck. I have limited range of motion in my head and neck because of fibrosis from the radiation; swallowing is somewhat of a challenge, and I have to be careful. But no one really sees it. It is not visible. I keep thinking these things are not as big a concern as they could have been, and I am alive. I look fine on the outside. I have just accepted it all. But then that is the type of person I am—logical, concrete, deal with what happens, do what I have to do.

And I learned I am far more resilient than I thought I was before this diagnosis. That was a surprise to me.

As far as advice to other patients I would just say, we all make choices. You can choose treatment and living, or you can choose not to have treatment. But treatment will mean there are things that will happen, and you will have to deal with them. You will have to almost disassociate yourself from your body at times.

### Linda

I'm 58 now and I was diagnosed with Stage 4 tongue cancer. November 2015 was when I was told I had cancer. January 14, 2016, I had surgery. I had a total glossectomy, reconstruction of my tongue, tracheotomy and a feeding tube. Five days after my first surgery I developed an infection and was brought back into surgery. This infection affected my new tongue and eventually it died.

I went through radiation and chemotherapy March 2016. Because I had radiation, I had to wait a year and a half before my doctor could attempt another reconstruction. So, for a year and a half I had no tongue. With all these changes I really had a lot of adjusting to do in my life.

I went back to work full time after 7 months. It definitely was an adjustment period for myself and my co-workers. I still had my tracheotomy in, so people at work were very curious of this.

I really didn't have problems communicating, I did a lot of writing, texting, and emailing. I was a Grocery Manager at our main store, and I did the ordering for one of our smaller stores. I had lots of interaction with suppliers and venders with no problems.

In September 2017 I had my second surgery to attempt reconstruction again. It was a success. After I healed, I spent a year and a half going to speech therapy. I am now able to have a conversation without writing on a piece of paper and I'm able to talk on the phone. My speech isn't 100% and it never will be; my speech is about 50%.

When I meet someone for the first time, I always try and put them at ease because some people are uncomfortable around people with disabilities. So, I always say to them, “If at any time during our conversation you don't understand me, please let me know.” This way they don't feel uncomfortable asking me to repeat myself. So many people say they understand what I'm saying but they don't, and that's very frustrating for me because they are nodding their heads but don't have a clue what I said. So that's why I always tell them upfront, just ask me to repeat myself if you don't understand.

One struggle for me at work was feeding. It was very awkward for me. This isn't something you see every day. Like, how many people do you know that are on a feeding tube? It was hard, but it got easier as time went by. I just said to myself, “I can't worry about what other people think.” So, when people would ask me about it, I would educate them.

My other struggle is how I look; I have no teeth. I have denture implants, but I can't wear them. I only use them for pictures. My tongue is very big, when I wear my dentures, I am not able to speak. So, with that being said, when people see me, they think I am a lot older than what I really am. My mouth is sunken, so I really struggle with how I look. I know that sounds vain, but your looks play a big part in getting a job in our society. So, my speech and no teeth are my struggles.

When the pandemic hit, like a lot of people, I lost my job. I have been applying for jobs for over a year now. I have over 30 years' experience in the grocery business. This is the one industry that thrived during pandemic. So, you would think that I wouldn't have a problem getting a job. Well, sad to say, no luck. They hear me speak and I never get a call. They aren't looking at my experience, they just hear me and see me. A lot of people think I'm deaf. They would be advertising that they are hiring right in front of store, but when I would ask them when I would drop off my resume, they would say, “No.” You don't realize how much discrimination there is until you are in that situation. I am definitely experiencing a lot of discrimination now.

One thing I'm proud of is I was able to help someone that was diagnosed with tongue cancer. He was struggling with if he should have the operation. We spoke and he was really worried about how he would sound. But once we spoke and he heard me, he decided to get the operation; he lost 50% of his tongue.

I wish I had been able to meet someone that went through this before I did, so I could ask questions about what to expect and how my life was going to change. It would put you at ease and you wouldn't feel like you were alone.

Stubbornness and attitude have gotten me through this. My goal was being able to carry on a conversation and I have accomplished this. You always go through some self-doubt. But that's when you get the attitude and stubbornness coming through, and have that “I don't care what anybody thinks; if they don't like me, that's their problem.” If I didn't tell myself this, I wouldn't be where I am today. It takes a long time to accept how you are, it's hard. But if you don't accept who you are, then it will be even harder to go through life.

### Leslie

Eighteen months after surgery for head and neck cancer, my face was still swollen. The scar, beginning behind my right ear and ending under my chin, was obvious to me. The skin was puckered and slightly red. I had no feeling on the right side of my face and neck with some muscle stiffness and range of motion difficulties. Facial nerve damage and numbness of my chin was juxtaposed with exaggerated pain at points on my lip. My voice sounded like that of a stranger. It was weak and lacked the intonation and diction necessary for conversation. When I tried to force my voice, it quickly became a whisper and then disappeared. I tolerated one brief activity only in a day. My physical condition made me feel even more isolated. Well-meaning acquaintances told me that I looked good or that radiation therapy was so much easier than chemotherapy. I felt wounded, weak and fragile. My face was completely different. I bought many scarves and collared shirts to hide my neck. But each evening I faced a mirror completing a teeth protocol that took 30–45 min. I had ample time to note the changes that cancer treatment had wrought. The mantra that reverberated constantly inside me was “damaged goods.”

I had lost 40 pounds. Physicians encouraged me to eat as much as I could. The radiation treatments destroyed my salivary glands meaning a painfully dry mouth, chronic irritation of oral tissues and difficulty swallowing with choking episodes from both liquids and solids. Baby pureed apple sauce added moisture to enable me to swallow more easily as liquids were simply swallowed and were ineffective. I had to physically work to swallow which quickly caused muscle fatigue and more difficulties. I would choke on dried saliva in my throat. It sounded like I was a heavy smoker. These episodes occurred frequently when I was trying to speak and eat at the same time. Do you ever get together socially when there is no food or drink? Each time I had to explain to strangers that cancer treatment caused my problems with speech, eating and the reason for carrying apple sauce. I could see they thought I was an alcoholic smoker, the typical head and neck patient profile, if they were health care professionals. For example, 9 years post my cancer a senior resident informed me a first diagnostic step was to determine my level of alcoholic liver damage. I was so diminished I could not respond before the resident left the room.

At a restaurant, I would search the menu for an item I might be able to eat. I then became one of those people querying the server about ingredients and asking for changes as I could not tolerate spices such as salt, pepper, ginger, or curry. Many spices created an overwhelming burning sensation in my mouth. Then, I had to ask about changing menu choices. “Could I have the fish with mashed potatoes as the rice it comes with is hard to swallow and makes me choke?” I would display my applesauce to back up my claims. These requests required a consultation with the chef, so my food was frequently delayed. The others had to then wait for me as I tried to eat. Food also stuck to my teeth without the lubrication of saliva. I would discover, for example, the food in my teeth when I went to the Ladies Room or returned home. I could hear “damaged goods” as I attempted to eat and tried to join the conversations. I quickly realized I could not do both so I would add an apology about my inability to speak when I was trying to eat. I would tell people I had already eaten or would eat later or would just order soup. The problem was the salt or spices that were already added made it more difficult to consume. I still felt it made others uncomfortable.

Fifteen years later, I struggle with many of the same issues. I could no longer meet the physical demands of work. I try to participate in a few team sport activities. My inability to speak clearly and to project my voice disadvantages our team. I feel bad about the extra work it creates for everyone in attempting to overcome my deficits. I continue one activity per day due to muscle fatigue of my neck and body. Thick tenacious secretions result in spontaneous choking episodes irrespective of food or drink intake. I must double swallow and ensure there are no crumbs in my mouth to ensure I don't choke if I look up. Again, my lack of saliva means everything sticks to my teeth. My swallowing difficulties are worse. I choke at least once during every meal. This disrupts all conversations and makes me the center of attention when I want to fade away. Added to this my lip numbness has progressed to drooling when attempting to swallow or spontaneously if I look down. This is hard to conceal from people around me. There is no quip from me to fix it. I will sometimes just say, “welcome to my world” but am mortified.

Head and neck cancer treatment changes everything, I feel. These changes are impossible to hide. They are literally in your face every moment of the day and night.

### Nancy

I was diagnosed with breast cancer 26 years ago when I was 36. It was a genetic cancer, but they had not yet found the marker at that time. They found it 7 years ago. It is BRCA 1, deletion of exons 18 and 19. I lost my mother, my grandmother, and a cousin, all within a two-year period to breast cancer.

Diagnosing my cancer was like looking for a needle in a haystack. When I went for a baseline mammogram, the technicians found something and called the radiologist. In a very short time, I was connected with a surgeon. In 2 weeks, he had done the biopsy and removed the tumor, and booked me for a double mastectomy. They took lymph nodes and one-third of the chest muscle, and fortunately nothing showed up in the nodes. I then had 6 months of chemo but no radiation.

We were worried that my chest would be concave with such radical surgery, so I was connected with a plastic surgeon about reconstruction. Temporary implants were put in during the surgery and I had regular appointments over the next year to expand the implants and tissue around them. Then permanent implants were put in at the end of that year. The two surgeons really worked well together and understood each other's goals—one to get all the margins clear and the other wanting as much tissue as possible to be left for the reconstruction. I was actually the first women to have implants put in at the time of the surgery in my province—my claim to fame!

A big worry was my arm and being able to move it afterwards. So, 10 days after surgery, I started physiotherapy and exercising to gain the best possible range of motion. I have not had lymphedema, although I have to be careful because I do not want it to develop—and that could happen anytime.

I had mouth sores as a result of the chemo and ended up losing the pigment on my upper and lower lips. Also, my eyelashes and half of one eyebrow did not grow back. But this was all easily resolved. I had tattoos done—and no one would ever know! It is amazing what can be done and looks so natural.

I also went through the change of life. I was on hormone replacements at the time of my diagnosis, as I had a hysterectomy because of endometriosis. This turned out to be an unknown lifesaving procedure as my ovaries were removed, thus eliminating the risk of ovarian cancer. I had real challenges with hot flashes. But thank goodness it was wintertime and all I did was open the back door!

One of the biggest problems was shaving under my arms. It is really hard to do when you can't feel it. I talked with a woman who had gone through the same thing, and she said, “You stand in front of a mirror and just make yourself do it.” It was good advice.

My husband was very supportive throughout. He said it made no difference to him how I looked after and that was important for me to hear. But my young daughter struggled, wondering if this was her future. I have lots of scars, but no one sees them. She really struggled with it all and that was hard, but we had amazing family support. The compassion and caring of my community made a real difference. That's what it is like in a small town.

I am happy my hair grew back in because I was told there was a 50% chance it would not. Although it is half as thick, that's OK, I had a lot before anyway. At the time, I wore a wig and got a special bra. But you have to laugh sometimes. I remember 1 day I was going into the post office, which is always busy. And it was windy. And the wind blew my wig backwards! What could you do but laugh and straighten it!

Reconstruction was not about getting back what I lost but more to do with looking and feeling normal again. Sure, they do a better job of the surgery now, but I am satisfied with the way I look. I tell other women when asked, it takes time to come to terms with a cancer diagnosis. It is a process and does not happen easily. Everyone's journey is their own and how they cope is unique. But we are strong, and the lucky ones survive.

### Sharon

I was diagnosed with uterine cancer. The D&C diagnostic was in September 2003, a hysterectomy in early January 2004, followed by an extended course of radiation therapy, and a hernia repair the next September. So, three general anesthetics in just under a year. I think the hernia started from throwing suitcases onto an airplane baggage belt. I realized later that probably was in the same category as the cautions not to vacuum or lift shopping bags after surgery, but that didn't occur to me at the time.

For me, physical changes from the surgery weren't the main issue. Friends and colleagues with medical experience remarked on how my mind was clearer and I had more energy than they expected. I had a desperate need to be back to normal and to get back to work. Granted I am self-employed, so there was the financial piece, but it was also about who I am and my personality. I had obligations to clients, and I was already frustrated. After the diagnosis I never was able to get a specific surgery date, or even a range of dates, so I could make plans with clients and family members who needed to make arrangements for international travel. And I was never able to get a clear sense of where the process was at—different parts of the system kept pointing the finger at other parts of the system to explain the delay or inability to tell me where things were at. Really makes you feel irrelevant.

I don't see how body image is separate from sense of identity as a whole person, and how I engage with the world. The main part of the puzzle for me was about sorting out my place in the world and way of being—more spiritual. And sorting through how I live confidently in the world afterwards—having had this kind of experience brings it home that life can change in an instant.

The loss of a uterus was not a big issue for me. People said the worst would be that it would catapult me into menopause and losing my identity as a woman, but I didn't find that to be the case. I had already seen a waning of my hormonal cycle where periods were few and scant. A lot I attributed to job stress. So, I felt I had sort of already gone through menopause. And I was at peace at being finished with my childbearing capacity.

In fact, there was one big blessing! A few months after surgery I was noticing a marked reduction in stamina, and sort of a generalized fatigue—maybe the benefit of the oxygen therapy I had after surgery was waning? It brought into focus a collection of cues and a diagnosis of sleep apnea. From the first night with a CPAP, my quality of sleep hugely improved!

For me the experience doesn't end with the time around the event. About 15 years later I became involved in some advocacy for patient-centered care and I found that all the recounting stories of my cancer experiences brought back so much. Once I realized what was happening, I think it really helped to process some of the emotions that I buried at the time.

One possible physical aspect was the gradual onset of incontinence I experienced. I learned recently that incontinence can be an after effect of having had a hysterectomy. No one had mentioned that before, and I was taken aback when I heard. I felt a sense of betrayal—why didn't' anyone tell me years ago? It would have been most beneficial to have known earlier, so I could take some action to slow or stop the progression. Maybe if it was a common thing, there'd be some research going on to develop something that could be done as a part of the surgical procedure to correct it. Maybe not, but I'd have liked to have that chance.

My orientation is pretty pragmatic. I like to have information. I understand life has challenges, and I expect to get quality information and a consistent story from skilled professionals, so I don't have to do all my own research. For many of us, it's part of feeling confident in the health care system. Examples like these erode our confidence in the health care system and in individual professionals.

In sum I'd say body image and identify is a lifelong affair one has with oneself. Information is influential and feeds into our identity, our sense of control. It is constantly in flux as we go through life. Each change helps us know more about who we are and how we engage with the world.

### Richard

I am a research scientist, who was diagnosed with prostate cancer 23 years ago when I was 52 years old. I promptly had surgery to remove my cancerous gland, followed by salvage radiotherapy to try and destroy residual cancer cells in the neighborhood where my prostate gland used to reside. For over 20 years I've been almost continuously on hormonal therapy, which is more properly called androgen deprivation therapy (ADT). ADT retards the growth of prostate cancer cells but doesn't necessarily kill them. Blood tests indicate that I have many quiescent cancer cells hiding in my body right now.

Before getting treated for prostate cancer, most of my research was with amphibians and had nothing to do with cancer. After going on ADT, I was so surprised by the side effects that I changed the direction of my research. Much of my work now relates to strategies for improving the quality of life of men treated for prostate cancer.

It was the impact of ADT on my body form and function which initiated the shift in my research. Indeed, it was the morphological effects that I experienced from ADT that led me to study the impact of cancer treatment on men's quality of life.

ADT has a slew of side effects, some of which are serious, but not seen. Others are seen, but not necessarily of medical concern. One of the most common side effects is increased adiposity, which is both serious and easy to see. It typically presents as increased fat in the abdominal region and thighs. Indeed, in my first 2 years on ADT, I experienced a ~10% increase in my body mass, all as fat.

Dr. Matthew Smith, who is an oncologist at Harvard, told me that when he asks his patients on ADT, “How are you doing?,” they often respond by saying, “Fine, except for this.” The “this” here is a specific gesture where they grab that new abdominal fat fold with both hands and jiggle it up and down. This action confirms the patients' awareness of how that additional abdominal fat has changed their body image.

That jiggle gesture is common among men on ADT, when discussing the side effects of their cancer treatment. I've made that gesture myself. Dr. Smith now calls the two-handed, abdominal jiggle a “positive Lupron sign” in reference to the historically most commonly prescribed drug for ADT.

One can gain weight as fat and not necessarily be distracted by it. My belly fat was not something that I constantly saw, sensed, and obsessed about. But after a year or so my ADT, I found myself very conscious of, and distracted by, the fact that the medial sides of my enlarged thighs were touching each other when I walked. This was a body image issue that was not necessarily conspicuous to others. But I felt it with every step I took after gaining that much weight on ADT. I have adapted to it now, and don't notice it as much. But after 20 years, I still struggle with keeping my BMI in a normal and healthy range.

And then there is gynecomastia. About 15% of the patients on ADT experience some breast development, which is influenced by the particular drugs they are on and how fat they are overall.

Some men are indifferent or unconcerned by gynecomastia, whereas others find it intolerable. One of my colleagues, who experienced it on ADT, had a mastectomy to remove the extra tissue. I alluded to how it affected me in an earlier essay (Wassersug, 2014), but, as a scientist I'm particularly interested in understanding why men vary so greatly in their tolerance for breast development. A colleague and I have previously suggested that gynecomastia may be more problematic for men who hold to a rigid gender hierarchy, with men dominant to women (Wassersug and Oliffe, 2009). That hypothesis has not been tested, but it might be helpful to know in advance of starting ADT, what are predictors of distress from gynecomastia. This could open the way to developing educational programs that might help reduce the distress and improve patients' quality of life in the long run.

There is one impact of ADT on body image that I do not believe has had much, if any, discussion in the literature. It's ADT's impact on one's hair. ADT causes a loss of body hair from the limbs and torso, but that doesn't seem to be of great concern to most patients on ADT, nor for that matter to their oncologists as it has no clinical significance. I've talked with patients, who were totally unaware of it. [However, one patient thought it malpractice that his doctor had not warned him about that.]

Hair on adult men's faces and head persists even when we are androgen-deprived. The persistence of hair of my head has had an unexpected impact on my body image—it makes me appear younger than my age. I know that to be true having asked many folks to guess my age. As long as I stay on ADT, I am unlikely to go bald.

Of course, how old one looks and how old one feels are not the same. I mention the topic of hair, however, just as a note that not all the ways the cancer treatments can impact body image are invariably negative.

## Concluding Remarks

The short narrations are personal reflections from the authors about their experiences with body image changes and the impact and challenges of living with those changes. There is a totality about the situations that defies “breaking it into pieces and isolating factors.” Experiencing and responding to bodily changes happens within, and is influenced by and intermingled with, the person's whole life. No two individuals have the exact same reaction and response, and the context of their daily living is an important influence. For some, there is a decided focus on the physical itself while others focus primarily on the emotionality which emerges in the situation; others simply focus on getting through each day with the demands the changes bring. There is a clear sense that body image is challenging for individuals to describe in isolation of its links with self-concept, self-identity, and self-esteem. Living with the day-to-day changes means living with the intermingled messiness—it is not about staying with strict conceptual definitions.

This article is offered as a way of illustrating a range of insights from individuals who have gone through an experience of cancer treatment and coped with bodily changes. The intention was to offer an avenue to reflect upon the individuality and the complexity of confronting body image changes resulting from that treatment. The personal descriptions present the voices of survivors and a glimpse into living with bodily changes. By reading the stories together, like looking at individual puzzle pieces and then putting the puzzle together, a picture emerges of the variation and complexity of living with body image changes from cancer and its treatment.

The changes and the responses to them range widely. For some the changes are profound and long lasting; for others, the body image concerns fade into the background and other issues take predominance. But what crosses the narratives, is evidence of a dynamic process unfolding as individuals live with the changes. We can see threads or storylines unfolding about how perspectives can change over time, the influence of information, the importance of relationships and support, and the personal discovery of resilience. There is an emphasis on the value of being alerted to possible changes ahead of time and learning what can be done about them. And there is illustration of how support from family, friends, and health care providers is significant and can make a difference for the individual coping with the change.

The notion of embracing storytelling to improve cancer care has been gaining ground in recent years (Atkinson and Rubindli, 2012). Perspectives from patients, survivors and family caregivers offer a rich base on which to launch conversations about necessary change within cancer care. Health care professionals and cancer system planners have begun to design and implement strategies to draw on patient stories for the purpose of incorporating them into all levels of health care including practice, education, research, and system planning (Bird et al., 2020).

At the practice or care delivery level, the individual practitioner can gain insight from hearing patient narratives which can be used in their own interaction with patients and families, enhancing their understanding of what is important to the individual and could be of assistance at the point of care. Two strategies leaders in clinical facilities have utilized to bring patient perspectives to the forefront of care delivery for health care professionals include implementing a person-centered philosophy across a health care facility (Health Quality Ontario, 2017) and introducing programs to screen for symptom and emotional distress (6th vital sign) (Bultz et al., 2011).

A person-centered philosophy challenges health care professionals to prioritize notions of dignity and respect, share information of relevance to the individual, engage with patients as partners, and facilitate patient collaboration in their own care (Throarinsdottir and Kristjansson, 2014; Calisi et al., 2016). Providing information and resource tools which enables individuals to be effective self-care agents is a growing expectation in practice (Howell et al., 2020) as cancer becomes more of a chronic type of illness and the number of survivors is increasing (McGeechan et al., 2018; Miller et al., 2019). Screening for distress programs introduce triage strategies using standardized distress screening tools at regular patient visits or specific points of transition in care when vulnerability may be more pronounced (Howell et al., 2012). The resulting information from patients can facilitate identification of any concerns which are of importance to patients and focus on those concerns as priorities for meaningful intervention (Howell and Olsen, 2011).

In the arena of education, patient narratives have been used to help students learn about patient and family experiences and health care professionals to gain insight about needed change in their care settings. Ultimately, the aim is to have practitioners be opened to listening to patients, giving time for individuals to share their stories, and being responsive to individualized concerns. Often, what is of concern or priority to a patient is not necessarily the same priority for the health care professional. As an example, a program designed by the Canadian Ovarian Cancer Association, entitled “Survivors Teaching Students,” was utilized at the University of Toronto to teach medical and nursing students about survivors' experiences (Fitch et al., 2011). Survivors were prepared to share their stories with classes of undergraduate students and respond to the student questions in small groups following the presentation. In another example, a theater play was designed for health care professionals to share the stories of cancer patients and family members and challenge their perspectives about the experience of being a cancer patient. The script was written as a series of vignettes based on results from qualitative research regarding living with breast cancer (Gray et al., 2000). The play was performed by survivors and delivered in cancer centers across Canada (Gray et al., 2003).

In terms of research, there is a growing trend toward embracing participatory action strategies or engaging patients and family members in research activities beyond being subjects in a trial or respondents to a survey (Canadian Institutes of Health Research, 2014; Hamilton et al., 2018). Engaging patients and family members as research team members or embracing approaches where patient advisory panels inform a research project design and measurement approaches have been utilized with success (Puts et al., 2017; Fitch et al., 2019). The participation aims to ensure the research focuses on questions of importance to patients and families, data collection approaches are respectful and appropriate, interpretation of results is done within a context of patient experiences, and dissemination reaches relevant audiences beyond health care professionals (Johnson et al., 2016; Bombak and Hanson, 2017).

Finally, use of strategies to engage patients and families as stakeholders in cancer system planning has been unfolding in some countries (International Alliance of Patients' Organizations (IAPO), 2007; National Health Service Department of Health, 2012; Staniszewska et al., 2014; Moody et al., 2016; Canadian Partnership Against Cancer, 2022). These strategies focus on intentionally hearing and using patient narratives in decision-making about new developments. The strategies have included analysis of both quantitative and qualitative data about what patients see as “quality” in cancer care (Institute of Medicine (United States) - Committee on Quality of Health Care in America, 2001; Darzi, 2008; Corner et al., 2013; Fitch et al., 2020) or suggestions they have about needed improvements (Nicoll et al., 2020; Fitch et al., 2021). Engaging individuals in co-design approaches (e.g., workshops, conferences, planning committees) and incorporating storytelling reflection has also been helpful for exploring patient perspectives and identifying priorities for improvement. Co-design approaches can be useful in planning new programs, developing new interventions, and developing survivor roles in communication and implementation of new approaches (Bethell et al., 2019).

In summary, hearing unique patient stories on an individual basis offers a variety of pictures and focus on various events and reactions. The stories offer the possibility of gaining new insights and understanding, new ideas for assessment or research, and new illustrations for professional, provider and/or patient education. Collectively they begin to illuminate underlying patterns that require change in moving toward a person-centric system that enables person-centered care (Cornish, 2020). Stories can be useful in the process of transforming an organization and gathering and mobilizing people for a common purpose. They can set the stage for co-creating or co-designing innovations in cancer care and finding meaningful ways to achieve a truly person-centered approach throughout the cancer care system.

## Data Availability Statement

The original contributions presented in the study are included in the article/supplementary material, further inquiries can be directed to the corresponding author at marg.i.fitch@gmail.com.

## Ethics Statement

Written informed consent was obtained from the individual(s) for the publication of any potentially identifiable images or data included in this article.

## Author Contributions

Each author contributed a story for inclusion. MF produced the initial draft and all authors edited until a final version was reached. All authors approved the final version.

## Conflict of Interest

SM is owner of the company Matthias Inc. AP is employed by YouByMia Photography. The remaining authors declare that the research was conducted in the absence of any commercial or financial relationships that could be construed as a potential conflict of interest.

## Publisher's Note

All claims expressed in this article are solely those of the authors and do not necessarily represent those of their affiliated organizations, or those of the publisher, the editors and the reviewers. Any product that may be evaluated in this article, or claim that may be made by its manufacturer, is not guaranteed or endorsed by the publisher.

## References

[B1] American Geriatrics Society Expert Panel on Person-Centered Care (2015). Person-centered care: a definition and essential elements. J. Am. Geriatr. 64, 15–18. 10.1111/jgs.13866 26626262

[B2] Atkinson S. Rubindli S. (2012). Narratives in cancer research and policy: voice, knowledge, and context. Crit. Rev. Oncol. Hematol. 84S2, S11–S16. 10.1016/S1040-8428(13)70004-0 23347413 PMC4241714

[B3] Bethell J. Puts M. T. E. Sattar S. Andrew M. K. Choate A. S. Clarke B.. (2019). The Canadian Frailty Priority Setting Partnership: research priorities for older adults living with frailty. Canad. Geriatr. J. 22, 1–11. 10.5770/cgj.22.336 31501680 PMC6707135

[B4] Bird M. Ouellette C. Whitmore C. Li L. Nair K. McGillion M. H.. (2020). Preparing for patient partnership: a scoping review of patient partner engagement and evaluation in research. Health Expect. 23, 523–539. 10.1111/hex.13040 32157777 PMC7321722

[B5] Bombak A. E. Hanson H. M. (2017). A critical discussion of patient engagement in research. J. Patient-Centered Res. Rev. 4, 39–41. 10.17294/2330-0698.1273 31413969 PMC6664365

[B6] Bultz B. Groff S. Williams A. Fitch M. I. (2011). Screening for distress, the 6^th^ vital sign: a canadian strategy for influencing the agenda for person-centered care. Psycho-Oncology 20 (Suppl. 2), P1-79, 239–240. 10.1002/pon.1932 21456060

[B7] Calisi R. Boyko S. Vendette A. Zagar A. (2016). What is person-centered care? A qualitative inquiry into oncology staff and patient and family experience of person-centered care. J. Med. Imaging Radiat. Sci. 47, 309–314. 10.1016/j.jmir.2016.08.007 31047255

[B8] Calman K. (2001). A study of storytelling, humour, and learning in medicine. Clin. Med. JRPL 1, 227–229. 10.7861/clinmedicine.1-3-227 11446621 PMC4951913

[B9] Canadian Institutes of Health Research (2014). Strategy for Patient-Oriented Research: Patient Engagement Framework, 1–11. Retrieved from: http://www.cihr-irsc.gc.ca/e/48413.html (accessed April 26, 2015).

[B10] Canadian Partnership Against Cancer (2022). Living With Cancer: A Report on the Paient Experience. Retrieved from: www.partnershipagainstcancer.ca (accessed March 2022)

[B11] Corner J. Wagland R. Glaser A. Richards S. M. (2013). Qualitative analysis of patients' feedback from a PROMs survey of cancer patients in England. BMJ Open 3, e002316. 10.1136/bmjopen-2012-002316 23578681 PMC3641435

[B12] Cornish P. (2020). Stepped Care 2.0: A Paradigm Shift in Mental Health. Berkeley California, CA: Switzerland Springer Nature (Apple Books).

[B13] Darzi A. (2008). High Quality Care for All: NHS Next Stage Review Final Report. London: Department of Health.

[B14] Eklund J. H. Holmstrom I. K. Kumlin T.. (2019). “Same or difference?” A review of reviews of person-centered and patient centered care. Patient Educ. Counsel. 102, 3–11. 10.1016/j.pec.2018.08.029 30201221

[B15] Fares J. Chung K. S. K. Abbasi A. (2021). Stakeholder theory and management: Understanding longitudinal collaboration networks. PLoS ONE 16, e0255658. 10.1371/journal.pone.0255658 34648505 PMC8516199

[B16] Fitch M. Macdonald C. Hutcheson K. A. McCulloch T. M. Martino R. (2019). Engaging stakeholders to identify relevant outcomes for dysphagia interventions. J. Support. Cancer Care 27(Suppl. 1), eP380: S168. 10.1007/s00520-019-04813-1 31102057

[B17] Fitch M. I. Coronado A. Schippka J. Chadder J. Green E. (2020). Exploring the perspectives of patients about their care experience: identifying what patients perceive as important qualities in cancer care. Support. Care Cancer 28, 2299–2309. 10.1007/s00520-019-05057-9 31478165

[B18] Fitch M. I. McAndrew A. Turner F. Ross E. Person I. (2011). Survivors teaching students: Increasing awareness about ovarian cancer. Canad. Oncol. Nurs. J. 21, 16–20. 10.5737/1181912x2111620 21462875

[B19] Fitch M. I. Nicoll I. Lockwood G. Newton L. Strohschein F. (2021). Improving survivorship care: perspectives of cancer survivors 75 years and older. J. Geriatr. Oncol. 12, 453–460. 10.1016/j.jgo.2020.09.012 32962951

[B20] Frank A. W. (2018). What is narrative therapy and how can it help health humanities? J. Med. Humanit. 39, 553–563. 10.1007/s10912-018-9507-3 29527629

[B21] Gray R. Sinding C. Ivonoffski V. Fitch M. I. Hampson A. Greenberg M. (2000). The use of research–based theatre in a project related to metastatic breast cancer. Health Expect. 3,137–144. 10.1046/j.1369-6513.2000.00071.x 11281920 PMC5080956

[B22] Gray R. E. Fitch M. I. Labrecque M. Greenberg M. (2003). Reactions of health professionals to a research based theatre production. J. Cancer Educ. 18, 223–229. 10.1207/s15430154jce1804_10 14766333

[B23] Hamilton C. B. Hoens A. M. Backman C. L. McKinnon A. M. McQuitty S. English K.. (2018). An empirically based conceptual framework for fostering meaningful patient engagement in research. Health Expect. 21, 396–406. 10.1111/hex.12635 28984405 PMC5750689

[B24] Health Quality Ontario (2017). Ontario's Patient Engagement Framework: Creating a Strong Culture of Patient Engagement to Support High Quality Health Care. Available online at: http://www.hqontario.ca/Portals/0/documents/pe/ontario-patient-engagement-framework-en.pdf (accessed June 26, 2018)

[B25] Howell D. Bultz B. Fitch M. I. Groff S. Williams A. (2012). Ensuring high quality response to screening for distress data. Oncol. Exchange 11, 2–8. Retrieved from: http://www.Oncologyex.com (accessed April 13, 2022).

[B26] Howell D. Mayer D. K. Fielding R.. (2020). Management of cancer and health after the clinic visit: a call to action for self-management in cancer care. JNCJ Natl. Cancer Inst. 113, djaa083. 10.1093/jnci/djaa083 32525530 PMC8096367

[B27] Howell D. Olsen K. (2011). Distress – the 6^th^ vital sign. Curr. Oncol. 18, 208–210. 10.3747/co.v18i5.790 21980246 PMC3185896

[B28] Institute of Medicine (United States) - Committee on Quality of Health Care in America (2001). Crossing the Quality Chasm: A New Health System for the 21st Century. Washington, DC: National Academies Press.

[B29] International Alliance of Patients' Organizations (IAPO) (2007). What is Patient-Centred Healthcare? A Review of Definitions and Principles. Available online at: http://iapo.org.uk/sites/default/files/files/IAPO%20Patient-Centred%20Healthcare%20Review%202nd%20edition.pdf (accessed June 26, 2018)

[B30] Johnson D. S. Bush M. T. Brandzel S. Wernli K. J. (2016). The patient voice in research—evolution of a role. Res. Involve. Engage. 2, 1–6. 10.1186/s40900-016-0020-4 29062507 PMC5611646

[B31] LeClair A. M. Kotzias V. Garlick J. Cole A. M. Kwon S. C. Lightfoot A.. (2020). Facilitating stakeholder engagement in early-stage translational research. PLoS ONE 15, eo235400. 10.1371/journal.pone.0235400 32614885 PMC7332000

[B32] Lowe D. Ryan R. Schonfeld L. Merner B. Walsh L. Graham-Wisener L.. (2021). Effects of consumers and health providers working in partnership on health services planning, delivery and evaluation. Cochrane Database Syst. Rev. 9, CD013373. 10.1002/14651858.CD013373.pub2 34523117 PMC8440158

[B33] McGeechan G. J. MCPherson K. E. Roberts K. (2018). An interpretative phenomenological analysis of the experience of living with colorectal cancer as a chronic illness. Clin. Nurs. 27, 3148–3156. 10.1111/jocn.14509 29752847

[B34] Miller K. D. Nogueira L. Mariotto A. B. Rowland J. H. Yabroff K. R. Alfano C. M.. (2019). Cancer treatment and survivorship statistics, 2019. CA Cancer J. Clin. 69, 363–385. 10.3322/caac.21565 31184787

[B35] Moody L. Nicholls B. Shamji H. Johnson N. Zwaal C. Stevens C.. (2016). Bringing person-centered care to practice with CCOès guideline for person-centered care in adult oncology services. J. Clin. Oncol. 34, 658. 10.1200/jco.2016.34.7_suppl.65

[B36] National Health Service Department of Health (2012). NHS Patient Experience Framework. Department of Health: London. Available online at: https://assets.publishing.service.gov.uk/government/uploads/system/uploads/attachment_data/file/215159/dh_132788.pdf (accessed June 20, 2018)

[B37] Nicoll I. Lockwood G. Chan R. J. Grundy P. Fitch M. I. (2020). What do adolescents and young adults perceive is the main challenge during the transition to survivorship? Canad. Oncol. Nurs. J. 30, 309–310. Retrieved from: http://canadianoncologynursingjournal.com/index.php/conj/article/view/1105 33165414 PMC7597769

[B38] Nolte E. Merkur S. Anell A. (Eds). (2020). Achieving Person-Centred Health Systems – evidence. Strategies and Challenges. Cambridge: Cambridge University Press. 10.1017/9781108855464

[B39] Puts M. T. E. Sattar S. Fossal T. Fitch M. MacDonald G. Hsu T.. (2017). The Senior Toronto Oncology Panel (STOP) study: research participation for older adults with cancer and caregivers. J. Natl. Compr. Canc. Netw. 15, 1208–1215. 10.6004/jnccn.2017.0159 28982746

[B40] Sharma T. Bamford M. Dodman D. (2016). Person-centered care: an overview of reviews. Contemp. Nurse. 51, 107–120. 10.1080/10376178.2016.1150192 26866531

[B41] Staniszewska S. Boardman F. Gunn L. Roberts J. Clay D. Seers K.. (2014). The Warwick Patient Experiences Framework: patient-based evidence in clinical guidelines. Int. J. Qual. Health Care 26, 151–157. 10.1093/intqhc/mzu003 24556816

[B42] Stover A. M. Strickler C. T. Hammelef K. Henson S. Carr P. Jansen J.. (2019). Using stakeholder engagement to overcome barriers to implementing patient-reported outcomes (PROs) in cancer care delivery – approaches from 3 prospective studies. Med. Care 57, S92–S99. 10.1097/MLR.0000000000001103 30985602 PMC12184201

[B43] Throarinsdottir K. Kristjansson K. (2014). Patients' perspectives on person-centered participation in health care: a framework analysis. Nurs. Ethics 2, 129–147. 10.1177/0969733013490593 23812560

[B44] Wassersug R. J. (2014). Prostate cancer, gonadal hormones, and my brain. J. Sex Marital Ther. 40, 355–357. 10.1080/0092623X.2014.921477 24846435

[B45] Wassersug R. J. Oliffe J. L. (2009). The social context for psychological distress from iatrogenic gynecomastia with suggestions for its management. J. Sex. Med. 6, 989–1000. 10.1111/j.1743-6109.2008.01053.x 19175864

[B46] Woods A. (2011). The limits of narrative: provocations for the medical humanities. Med. Humanit. 37, 73–78. 10.1136/medhum-2011-010045 22038696 PMC4281385

